# Flapless Decoronation: A Minimally Invasive Approach

**DOI:** 10.3390/ijerph20010603

**Published:** 2022-12-29

**Authors:** Boaz Shay, Eitan Mijiritsky, Meital Bronstein, Mor Govani-Levi, Tal Ben Simhon, Tali Chackartchi

**Affiliations:** 1 Faculty of Dental Medicine, Hebrew University of Jerusalem, Jerusalem 9112102, Israel; 2 Department of Endodontics, Hadassah Medical Center, Jerusalem 9112102, Israel; 3 Department of Head and Neck and Maxillofacial Surgery, Tel-Aviv Sourasky Medical Center, The Sackler Faculty of Medicine, Tel-Aviv University, Tel Aviv 6139001, Israel; 4 The Maurice and Gabriela Goldschleger School of Dental Medicine, Tel Aviv University, Tel Aviv 6139001, Israel; 5 Department of Periodontology, Hadassah Medical Center, Jerusalem 9112102, Israel

**Keywords:** dental trauma, decoronation, flapless decoronation, ridge preservation, minimally invasive

## Abstract

Traumatic injuries to the permanent dentition are most common in children. In severe dentoalveolar injuries, especially avulsion and intrusion, dentoalveolar ankylosis is a common complication, leading to adverse effects on the developing alveolar bone and interfering with the eruption of the adjacent teeth. The decoronation procedure was suggested in 1984 to reduce these side effects related to ankylosis. The objective of the current publication is to describe a minimally invasive, flapless decoronation procedure aimed to minimize and simplify the surgical procedure of decoronation, and ease its clinical acceptance, particularly in young children. The technique is described in a detailed protocol and demonstrated in two cases. Under local anesthesia, the dental crown is removed, and the root is reduced by 1.5–2.0 mm apically to the marginal bone crest. The root canal content is then removed, allowing it to fill with blood. The socket is coronally sealed with a porcine-derived collagen matrix (PDCM) sutured using the “parachute” technique over the resected root, allowing close adaptation to the surrounding soft tissue. In conclusion, the presented technique of flapless decoronation is a modification of the classic decoronation procedure, which can be used as a minimally invasive technique to simplify the surgical procedure and the post-operative process.

## 1. Introduction

Traumatic injuries to the permanent dentition are most common in children 8–12 years of age, with a prevalence of 22% [1]. Of these, 0.5–3.0% [2] are avulsions and 0.5–1.9% are intrusive luxation [3,4]. Overall, avulsion of the permanent teeth is seen in 0.5–16% of all dental injuries [5]. A common complication after avulsion and intrusion injuries is dentoalveolar ankylosis, in which the alveolar bone fuses to the root substance. An ankylosed root is gradually resorbed and replaced by bone [5,6,7]. The clinical signs of ankylosis are a high-pitched (metallic) percussion sound and decreased mobility followed by increasing infraposition and tilting of adjacent teeth in growing individuals. The condition in children is progressive, and the radiographic appearance depends on the progress, ranging initially from loss of the lamina dura to root replacement, eventually resorbing the entire root [6].

This condition is progressive, and the rate of resorption seems to vary with age [8]. Ankylotic teeth pose a problem if present during the growth spurt. These teeth do not erupt simultaneously with the alveolar bone, which grows downwards and anteriorly in the maxilla [6,9]. The discrepancy between the growth of the alveolar bone process around normal teeth and ankylosed teeth results in the clinical appearance of infra-occlusion. In 1984, the decoronation technique was introduced by Malmgren et al. [10], aimed at removing the ankylotic tooth crown in young, growing individuals, while preserving the alveolar ridge and improving conditions for a subsequent prosthetic therapy that would restore the missing tooth. In decoronation, the clinical crown is removed as well as the root filling while maintaining the resorbing root as a matrix for new bone apposition. This technique was based on studies that had shown new marginal bone forming coronal to the surface of submerged roots covered with a mucoperiosteal flap [11,12]. However, in order to submerge the decoronated root and the surrounding bone, two vertical releasing incisions are advised, to facilitate the mobilization and coronal advancement of the flap [10]. This soft-tissue manipulation should be handled with care in order to prevent scar formation, especially if performed in the esthetic zone. These flap manipulations might also result in post-operative swelling and patient discomfort.

In the current publication, we suggest a flapless procedure aimed to minimize and simplify the surgical procedure of decoronation, mostly performed in young children. The technique will be described and then demonstrated with two cases.

## 2. Materials and Methods

### 2.1. Description of the Technique

#### 2.1.1. Ethical Considerations

The present publication is a demonstration of the technique using the presentations of two cases and therefore does not require IRB approval. Nevertheless, the procedures were conducted in accordance with the 1964 Declaration of Helsinki, and the patients’ parents gave informed consent to the procedure.

#### 2.1.2. Indications and Contra-Indications for Decoronation

The indications and contra-indications for flapless decoronation are as described by the International Association of Dental Traumatology (IADT), which recommends decoronation in growing patients to treat an ankylosed tooth, to minimize the consequences of infraposition when the ankylosed tooth shows evidence of infra-occlusion that is considered esthetically unacceptable, and a simple restorative solution cannot correct it [5]. In addition, according to Malmgren, the timing and indication for decoronation in ankylotic teeth in growing patients are as follows:

- Age 7–10 (early mixed dentition)—decoronation should be performed within two years.
- Age 10–12 (late mixed dentition)—requires individual monitoring, since a rapid infraposition rate is expected when the patient reaches the pubertal growth spurt; decoronation is indicated upon infraposition diagnosis.
- In later stages (early permanent dentition)—the infraposition rate might be slow, not necessarily requiring decoronation; nevertheless, annual follow-ups should be carried out [13,14,15,16].

#### 2.1.3. Clinical Procedure

**Figure 1:**
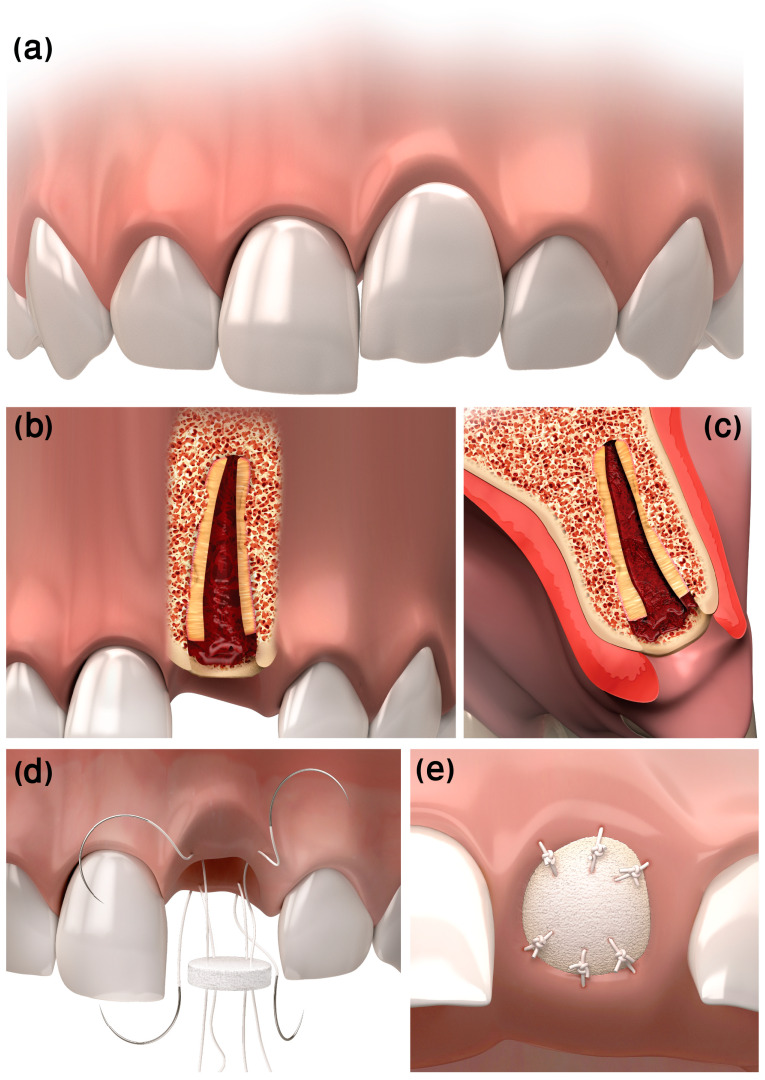
Flapless decoronation illustration. (**a**) Pre-op ankylotic infrapositioned left central incisor. (**b**) Coronal and (**c**) sagittal cross-sections of the resected ankylotic root below the marginal bone. Blood is filling the canal space and clotting coronal to the resected root. (**d**) “Parachute” technique suturing. (**e**) Post-op final suturing of the Mucograft^®^ Seal.

Following local anesthesia infiltration (Lidocaine 2% with epinephrine 1:100,000, USP, Henry Schein^®^ Dental, Melville, NY, USA), the dental crown is removed, and the root is reduced by 1.5–2 mm apically to the marginal bone crest, using a round diamond burr. The root canal content is then removed using rotatory instruments such as Gates Glidden drills (Mani^®^, Tochigi, Japan) and K-files, and the canal is thoroughly irrigated with saline solution, subsequently allowing it to fill with blood (Figure 1b,c). To seal the socket coronal to the resected root from the oral cavity, a bi-layer (compact and spongy) round porcine-derived collagen matrix (PDCM), i.e., Mucograft^®^Seal (Geistlich Pharma AG, Wolhusen, Switzerland), which is a newly developed alternative to Subepithelial connective tissue graft (SCTG) for soft-tissue regeneration [17], is used. The Mucograft^®^Seal is adapted with 5/0 coated VICRYL^®^ (polyglactin 910) sutures (Ethicon Inc., Somerville, NJ, USA), using the “parachute technique” [18] as follows: 4 separate sutures are inserted through the Mucograft^®^Seal, from the compact into the spongy layer at the mesiobuccal, distobuccal, mesiopalatal, and distopalatal corners of the round PDCM, going through the soft tissue from inside the socket outward at the matching papilla (Figure 1d). All 4 threads are pulled at once to parachute the Mucograft^®^Seal into the socket opening, over the resected root, allowing close adaptation to the surrounding soft tissue. The sutures are tied at all matching points, and additional buccal and lingual sutures are inserted for closer adaptation and stabilization of the graft (Figure 1e).

Post-op instructions include a soft, cold-to-lukewarm diet for 24 h; a soft diet for at least 2 weeks; avoiding pressure on the graft for 6 weeks (to prevent rapid resorption of the graft); meticulous oral hygiene, including daily anti-bacterial mouth rinse; and comprehensive follow-ups.

## 3. Technique Demonstration

### 3.1. Case 1

- Dental history

**Figure 2:**
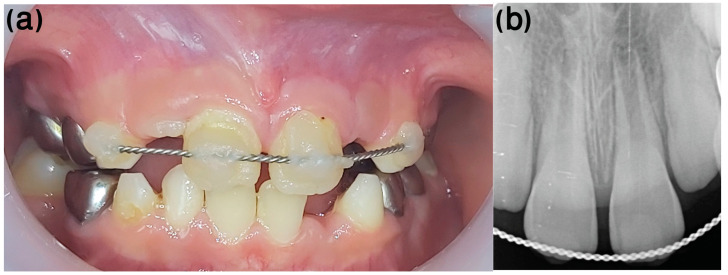
Case 1, one week post-injury. (**a**) Clinical and (**b**) radiologic appearance one week following avulsion, re-implantation, and splinting of tooth #21.

A 9-year-old healthy male patient was referred to the Endodontic Clinic at the Hadassah Medical Center for treatment of the left maxillary central incisor (tooth 21, according to FDI notation). Nine days earlier, he fell off his bike, causing avulsion of tooth 21. The tooth was kept dry for more than 90 min. However, despite the poor prognosis, re-implantation was conducted and the tooth was splinted (Figure 2a).

**Figure 3:**
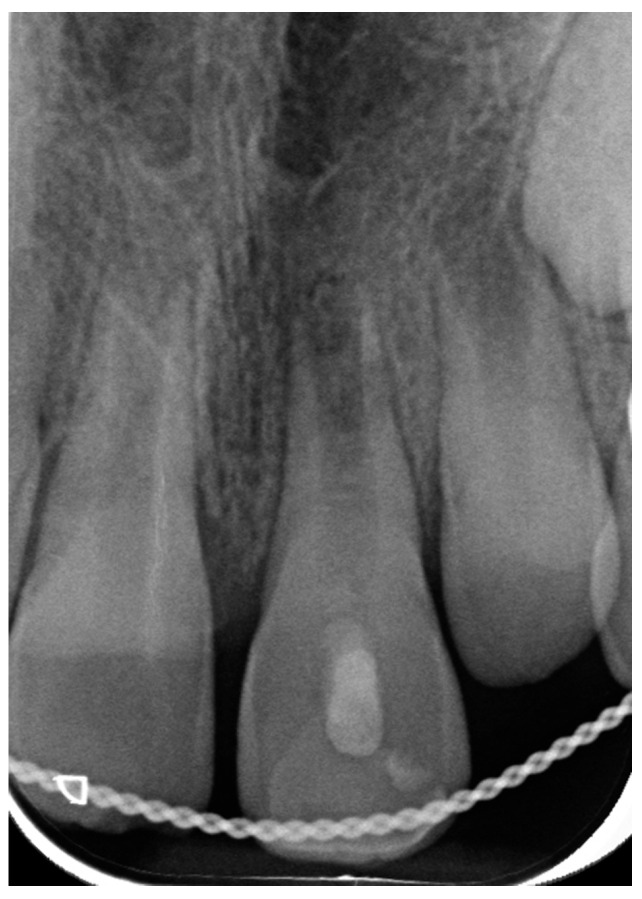
Case 1, one month following avulsion. Radiographs exhibited initial evidence of inflammatory resorption. Clinically, a metal sound in percussion with no detectable mobility suggested the tooth was ankylotic.

At the first appointment, clinical examination revealed that the tooth had no discoloration. No soft tissue or lacerations were observed, and radiographs showed immature roots (Figure 2b). Therefore, root canal treatment was conducted in which the necrotic pulp tissue was removed, and the root canal space was filled with a medication of calcium hydroxide. One month after the avulsion, a metal sound in percussion was noted in the clinical examination, with no detectable mobility, suggesting the tooth was ankylotic. Radiographs taken at this stage exhibited evidence of inflammatory resorption (Figure 3). We then re-accessed the root canal and refreshed the medicament.

**Figure 4:**
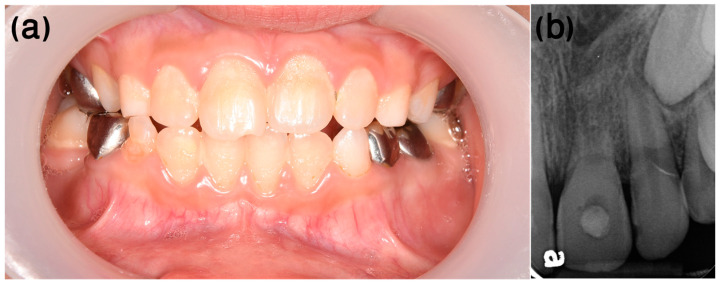
Case 1, four-month follow-up. (**a**) The infra-occluded appearance of the tooth can be noted. (**b**) Root replacement resorption was progressing (the white rotated symbol “a” in the bottom left corner of the figure is part of an embedded symbol in the phosphorus plate by the manufacturer for orientation).

After several follow-up appointments, every 3–4 months, including clinical and radiological assessments, the infra-occluded appearance of the tooth was noticed (Figure 4a). Furthermore, excessive root replacement resorption was observed (Figure 4b). These follow-up examinations included renewing the intracanal calcium hydroxide dressing.

Following careful analysis of the case by an orthodontist, a periodontist, and an endodontist, the suggested treatment plan included decoronation of tooth 21, followed by orthodontic treatment. A space maintainer that contained a temporary tooth was planned to preserve the esthetic appearance. The patient’s parents gave informed consent to the procedure.

- Clinical procedure

**Figure 5:**
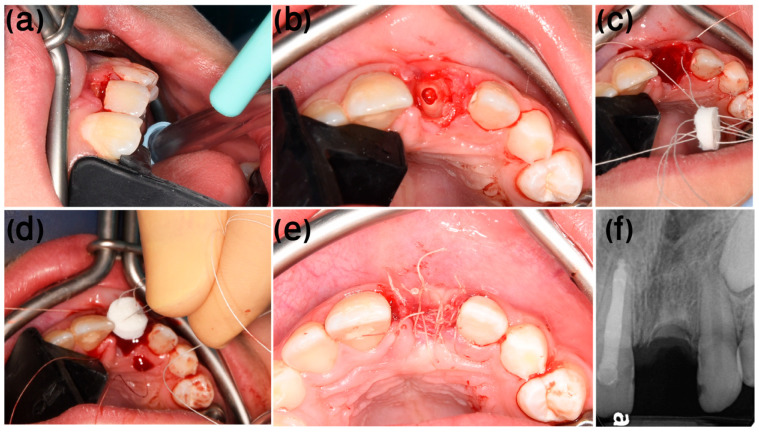
Case 1, flapless decoration procedure. (**a**) The crown of the tooth was separated from the root, 2 mm below the cementoenamel junction, using a diamond bur. (**b**) Occlusal appearance of the root after crown removal. (**c**) A round PDCM was adapted with 5/0 coated VICRYL^®^ (polyglactin 910) sutures, implementing the “parachute technique”. (**d**) Sutures were tightened simultaneously in all aspects, for ease of manipulation and adaptation. (**e**) Additional simple interrupted bucco-palatal sutures were added as needed for stabilization of the graft. (**f**) Post-op radiograph (The white rotated symbol “a” in the bottom left corner of the figure is part of an embedded symbol in the phosphorus plate by the manufacturer for orientation).

After infiltration of local anesthesia (2% lidocaine; 1:100,000 epinephrine, Henry Schein^®^, NY, USA), the dental crown was separated from the root, 2 mm below the marginal bone, using a round diamond bur (Figure 5a). The root canal content was excavated using a Gates Glidden no. 3 drill (Mani^®^, Tochigi, Japan), followed by K-files no. 60 and no. 70 (Mani^®^) (Figure 5b). Subsequently, root canals and the surrounding tissues were irrigated with saline solution. The procedure was considered complete when the root canal was filled with blood. To protect the root canal from the oral cavity, a round PDCM (Mucograft^®^Seal) was adapted with 5/0 coated VICRYL^®^, using the “parachute” technique [18] (Figure 5c–e). A space maintainer containing a provisional tooth was adapted. Post-operative medication included a chlorhexidine 0.2% mouth wash twice daily for two weeks, and one post-operative dose of NSAIDs to control post-operative pain.

- Follow-up

**Figure 6:**
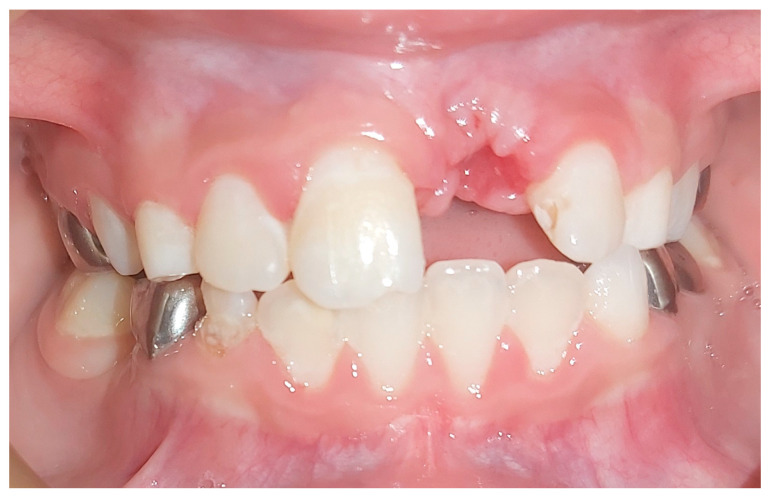
Case 1, four weeks post-surgery. Four weeks after the flapless decoronation procedure, sutures were removed, and epithelization of the treated area was noted.

**Figure 7:**
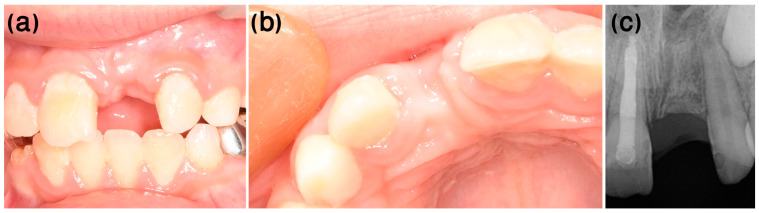
Case 1, six-month post-surgery follow-up. (**a**,**b**) Ridge dimensions can be noted, as well as (**c**) the radiographic appearance of bone apposition coronal to the resected root and replacement resorption progression.

**Figure 8:**
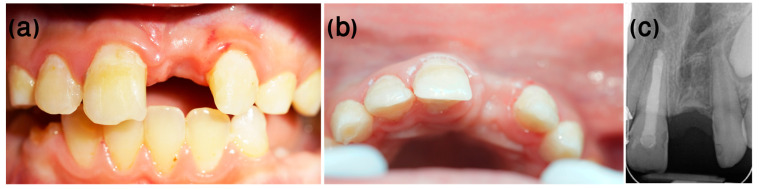
Case 1, one-year post-surgery follow-up. (**a**,**b**) Clinical appearance. (**c**) Replacement resorption progression and bone apposition coronal to the resected root are evident.

The patient reported no pain or post-operative extra-oral swelling (Figure 6). Six months post-operatively, the tissue continued to mature (Figure 7a,b). The radiologic and clinical appearance at the 6-month (Figure 7) and 1-year (Figure 8) post-op follow-ups indicated replacement resorption progression, together with bone apposition coronal to the resected root. 

### 3.2. Case 2

- Dental history

**Figure 9:**
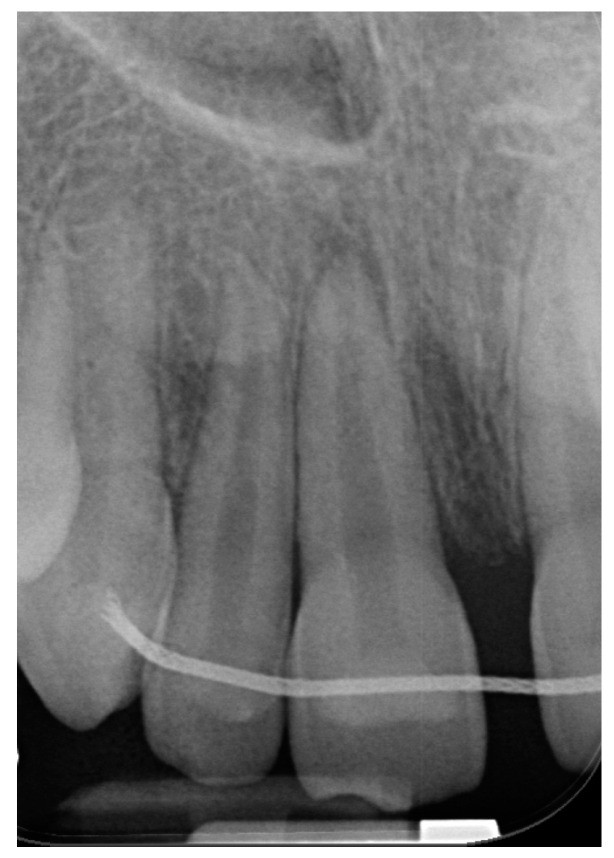
Case 2 radiograph post-replantation. Tooth #11 was re-implanted and fixated after evulsion.

A 12-year-old boy was referred to the Endodontic Clinic at the Hadassah Medical Center for treatment of the right maxillary central incisor (tooth 11 according to FDI notation). Fourteen days earlier, the boy had a bicycle accident resulting in tooth #11 avulsion. The tooth was stored in milk for 3 h prior to replantation and fixation (Figure 9).

**Figure 10:**
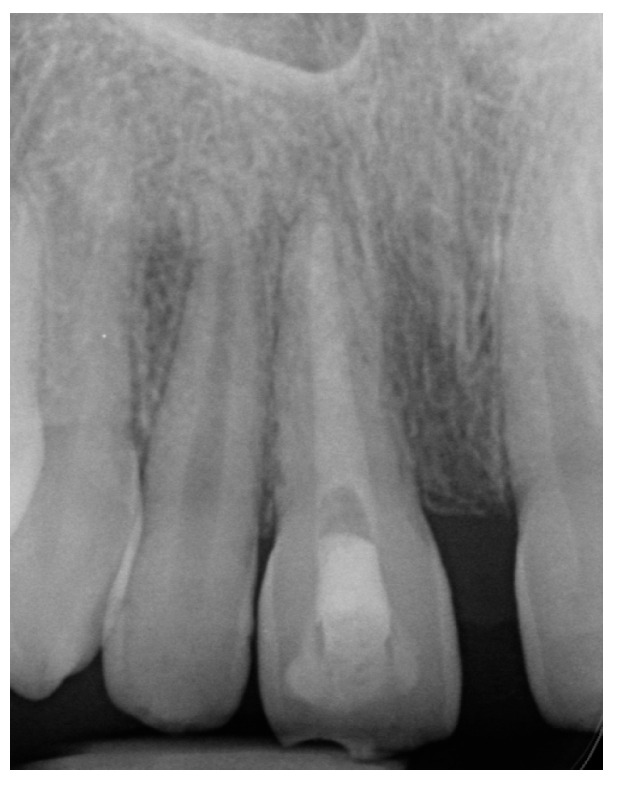
Case 2, two months post-injury. Two weeks after the trauma, root canal treatment was performed, and at the two-month post-injury examination, ankylosis was already diagnosed.

At the first appointment, clinical examination disclosed class 1 mobility according to Miller’s classification [19] and sensitivity to percussion. Therefore, root canal treatment was performed, initially using a calcium hydroxide dressing. At the two-month examination, tooth #11 demonstrated a high-pitched percussion sound and decreased mobility, the radiograph showed primary signs of replacement resorption, and ankylosis was diagnosed (Figure 10). 

**Figure 11:**
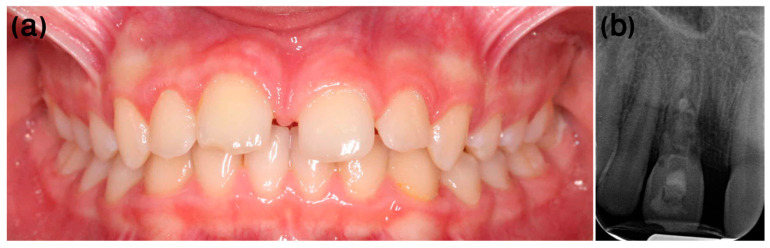
Case 2, one-year follow-up. (**a**) One year after the trauma, infra-occlusion of 2 mm was evident. (**b**) Marked replacement resorption was noted on the X-ray.

One year after the dental trauma, infraposition of 2 mm was evident (Figure 11a), and marked replacement resorption was noted on the X-ray (Figure 11b). At this stage, the treatment plan included decoronation of tooth #11, scheduled according to the commencement of orthodontic considerations and treatment.

The patient’s parents gave informed consent to the procedure.

- Clinical procedure

**Figure 12:**
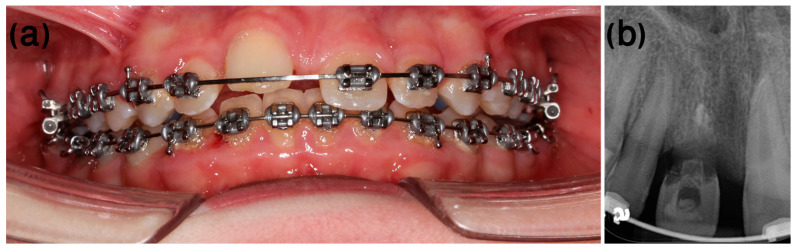
Case 2, day of decoronation, pre-op. (**a**) Clinical and (**b**) radiographic appearance on the day of decoronation, prior to the procedure (the white rotated symbol “a” in the bottom left corner of the figure is part of an embedded symbol in the phosphorus plate by the manufacturer for orientation).

**Figure 13:**
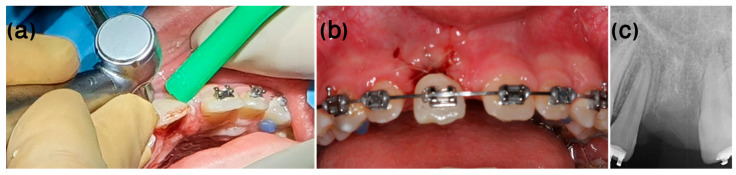
Case 2, flapless decoronation procedure. (**a**) Removing the clinical crown using a tungsten bur. (**b**) Coated VICRYL^®^ (polyglactin 910) sutures were used to stabilize the PDCM over the exposed root. (**c**) Radiographic appearance at the end of the procedure.

Orthodontic treatment was initiated, and decoronation was scheduled for 8 months later (Figure 12). The flapless decoronation procedure was implemented as described in Case 1 (Figure 13a–c).

- Follow-up

**Figure 14:**
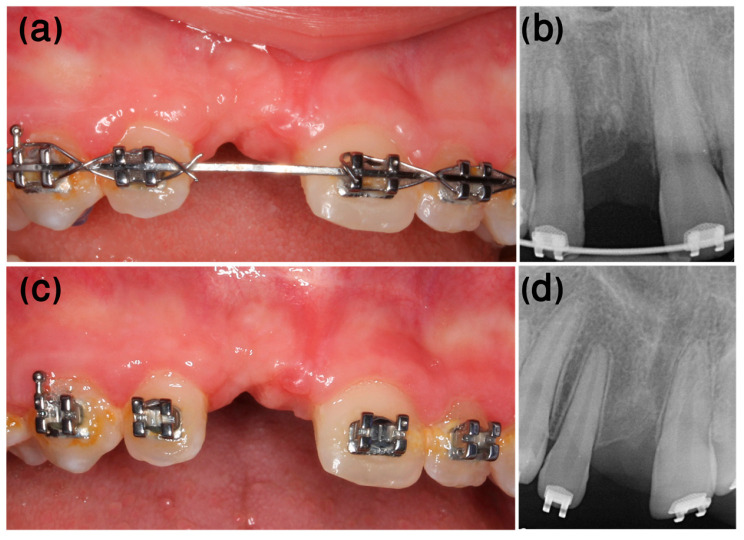
Case 2, post-surgery follow-ups. (**a**,**b**) Six-month follow-up. (**c**,**d**) One-year follow-up.

As in the first case, the radiologic and clinical follow-ups 6 months and 1 year after decoronation (Figure 14) demonstrated replacement resorption progression, together with bone apposition coronal to the resected root.

## 4. Discussion

This article presents a minimally invasive, flapless modification of a currently practiced technique of decoronation using PDCM. While the technique itself has not been reported before (use of PDCM for decoronation), PDCM has been used in similar clinical scenarios (extraction sockets, gingival augmentation, etc.) [17,20]. Although several publications presented some modification of the decoronation procedure, especially without vertical incisions, all of them included a mucoperiosteal flap [21,22,23,24]. In the presented technique, we sought to eliminate the mucoperiosteal flap, in order to allow a minimally invasive, faster, and more comfortable decoronation procedure for the child and parents, as well as potentially easier post-operative healing and better patient and parent compliance with the procedure. Using the flapless decoronation technique, we observed bone apposition coronal to the resected root, suggesting this technique preserves the alveolar ridge. Indeed, previous studies have shown that this process of bone healing enables the preservation of the vertical dimension of the ridge during eruption and development [6,10,25]. To the best of our knowledge, there is no report in the English literature of a flapless decoronation.

Decoronation of infrapositioned ankylosed teeth is considered the gold standard treatment for young growing patients [26] and is a well-established, efficient, and predictable approach aimed at preserving the alveolar bone in children who are still facing facial growth in order to prevent bone deformations in cases of ankylotic teeth [16], while most alternative techniques, such as surgical luxation, orthodontic distraction, and auto-transplantation, have been associated with an unpredictable prognosis in ankylotic teeth [25].

In general, it is recommended to remove an ankylosed tooth before severe infra-occlusion and tilting of neighboring teeth development [27], which may result in unsatisfactory esthetic consequences [24]. However, ankylotic tooth extraction will be followed by significant dimensional changes to the alveolar ridge contour [28,29,30,31,32]. The mean resorption in the height and width of the alveolar ridge may reach approximately 50% and occurs predominantly within the first 3–6 months [32]. These dimensional changes are expected to be more eminent in cases of traumatic extraction of ankylotic teeth. By employing decoronation for the extraction of ankylotic teeth, the alveolar width is preserved and the lost vertical bone of the alveolar ridge in these growing individuals is rebuilt [16]. This will later allow an improved restoration of the missing tooth in terms of esthetics, phonetics, and periodontal maintenance [33].

The original decoronation technique, introduced by Malmgren et al. [10], advises submergence of the exposed bone and root by the coronal advancement of a mucoperiosteal flap. Flap mobilization is enabled using two vertical releasing incisions (VRIs), one on each side of the involved tooth. It is well established that VRIs might damage the lateral blood supply to the flap, resulting in unesthetic, visible white scars (keloids) [34,35]. VRIs might also result in a greater incidence of swelling, pain, and bleeding [36], possibly leading to higher patient morbidity in mucogingival surgeries [37]. The presence of sutures in the apical region of the lining mucosa may contribute to an unpleasant post-operative course in patients treated with VRIs. Therefore, VRIs are avoided whenever possible in mucogingival surgery [37]. Moreover, flap elevation and coronal advancement of a full-thickness flap might cause post-operative bone resorption, marginal recession at the adjacent teeth, impaired healing of the papillae, and loss of keratinized mucosa [38].

The importance of a minimally invasive technique is particularly important to achieve better patient experience. It is well known that traumatic experience for a young child may affect their future cooperation during dental treatments [39,40]. Young patients who experience dental trauma are required to undergo several invasive dental and medical procedures. The study by Zhang et al., evaluating the psychological impact of decoronation in children and adolescents, demonstrated that child fear of the treatment had the highest score among the major causes of stress and its severity while choosing to proceed with decoronation [24]. 

To achieve the goal of the suggested technique, we used a round porcine-derived collagen matrix (PDCM), i.e., Mucograft^®^Seal, primarily introduced for the treatment of post-extraction alveolar ridge preservation [20]. The seal is a small, round modification of PDCM and is designed to seal sockets with preserved buccal walls following ridge preservation with bone grafts. The PDCM is easy to handle and does not require treatment or hydration prior to placement [20]. The use of PDCM and bioabsorbable collagen wound dressing has been proven to form an effective barrier for the bone graft in alveolar ridge preservation procedures [41,42]. Although exposure of the PDCM to the oral environment might result in a faster degradation rate, no adverse effects are found if these barriers are left exposed. Through the use of PDCM as a coronal seal, both the decoronated root and the exposed bone can be isolated from the contaminated oral cavity. It is speculated that the PDCM serves as a scaffold and allows the growth of an epithelium that is derived from the surrounding keratinized tissue [43]. The PDCM’s spongy portion is designed to promote cellular ingrowth; this promotes neovascularization and wound healing, resulting in enhanced root attachment and gingival thickness [17,44]. Multiple sutures were used, primarily to stabilize the barriers in place [41], in a manner similar to the suturing method called the “parachute” technique, primarily invented for cardiovascular valve surgeries [45] and later modified for and adopted in other medical procedures [46,47], including in maxillofacial surgery, as described by Liu et al. [18]. 

### Strengths and Limitations

The main strength of the presented technique is the minimally invasive procedure, and the possibility of avoiding post-surgical complications related to flap elevation. The advantages of the minimally invasive technique become all the more important since decoronation is mostly performed in young children. While the minimally invasive technique is mainly an advantage, it also has limitations such as limited fields of view of the surgical site, and the learning curve needed to acquire the skills for the manual handling of the new technique.

A limitation of the current publication is that quantitative 3D measurements of bone gain were not included; future studies should include them using a proper radiological technique with minimal ionizing radiation and ethical consideration in accordance with the ALARA principles for pediatric populations.

Further studies are needed to evaluate the predictability of the clinical outcome of this procedure, including clinical parameters such as post-operative swelling, ridge alternations, and long-term soft-tissue dimensions. 

## 5. Conclusions

The presented technique offers a surgical modification of the classic decoronation procedure presented by Malmgren et al. [10] in 1984. By avoiding flap elevation and soft-tissue manipulation, we can expect ridge preservation and an easier post-operative process for the patient. 

## Data Availability

The data presented in this publication are available on request from the corresponding author. The data are not publicly available due to privacy restrictions.
